# Clinical Challenges and Surgical Interventions in Managing Neck Hematoma After Cervical Spine Fusion: A Case Report

**DOI:** 10.1155/2024/3173782

**Published:** 2024-10-04

**Authors:** Parimal Rana, Justin Turcotte, Sohail Zaidi

**Affiliations:** Luminis Health Orthopedics at Anne Arundel Medical Center, 2000 Medical Parkway, Suite 503, Annapolis 21401, Maryland, USA

## Abstract

This case study discusses a 47-year-old Caucasian male with a past medical history of dyslipidemia, gastroesophageal reflux disease, previous cervical spine surgery, and anxiety who developed a neck hematoma postrevision of a C5–6 cervical spine fusion. Emergent neck exploration and evacuation of the hematoma were performed, and ventilation was restored. The patient was transferred to the intensive care unit and extubated on postoperative day 5 with a stable wound and no residual bleeding. At the 3-week follow-up appointment, the patient was noted to be doing well, with a chest radiograph showing no effusion or hematoma. This report elucidates the challenges posed by acute clinical symptoms and their correlation with the underlying cause, as well as the subsequent management and outcomes of a neck hematoma complication following cervical spine surgery.

## 1. Introduction

Anterior cervical spine (ACS) surgery, a common procedure performed by orthopedics and neurosurgeons, typically yields favorable clinical outcomes [1, 2]. However, complications, particularly hematoma formation causing airway obstruction, remain a significant concern, with a reported incidence rate ranging from 0.2% to 1.9% [3–5]. These hematomas can be categorized into wound hematoma, spinal epidural hematoma, and retropharyngeal hematoma, with the latter posing a higher risk due to its potential to compromise the airway [6]. In fact, postoperative cervical hematoma has been associated with a significant increase in mortality rates [7]. Additionally, it is noteworthy that the prevalence of cardiac complications in ACS surgery is relatively low, with only 4 out of 1000 cases (0.004%) experiencing such issues [8]. Therefore, surgical trauma–related complications, such as hemorrhage and hematoma formation, should not be overlooked or overshadowed by cardiac concerns.

## 2. Case Presentation

### 2.1. History

The patient, a 47-year-old Caucasian male, had a pertinent medical history, including dyslipidemia, gastroesophageal reflux disease, and anxiety. He had previously undergone C5–6 anterior fusion in 2017 due to cervical spondylosis with radiculopathy, with subsequent axial spine discomfort. The decision was made to revise the surgery of the fusion with an additional extension to the C6–7 level, and the procedure was performed without intraoperative complications. Postoperatively, after approximately 4 h, the patient developed sudden-onset chest pain and diaphoresis while being prepared for discharge from the postanesthesia care unit. Suspecting cardiac ischemia, blood labs were drawn, and a chest radiograph was completed within the hour. However, the first physical exam of the neck was not completed until 2 h after symptom onset, which revealed a large neck hematoma. This prompted an urgent consultation by the floating anesthesia care team to protect the airway. The operating neurosurgeon was contacted, and cardiothoracic and vascular surgery were consulted in the operating room. Due to the elapsed time from the onset of symptoms to the evaluation of the neck swelling, a large hematoma continued to expand, compressing the patient's trachea and requiring emergent surgical intervention.

### 2.2. Physical Exam

Upon examination, the patient presented with a seal-like cough, bilateral wheezing on lung auscultation, and a large swelling on the left side of the neck. Vital signs revealed elevated blood pressure (140/80 mmHg, subsequently raised to 190/135 mmHg), bradycardia (49 beats/min), normal respiratory rate (14 breaths/min), and oxygen saturation of 97%–100% on 2 L O_2_.

### 2.3. Investigations

**Figure 1:**
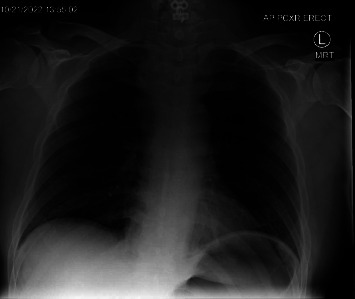
Initial cervical fusion postoperative chest radiograph.

**Figure 2:**
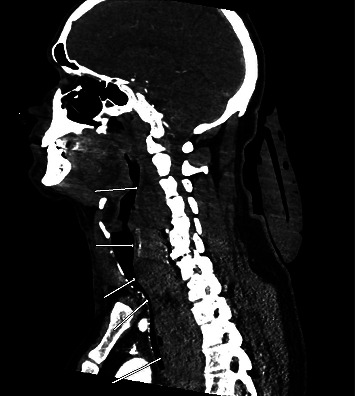
CT image demonstrating a large prevertebral soft tissue hematoma extending from C3 through the carina.

**Figure 3:**
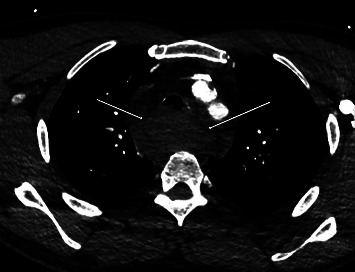
CT image demonstrating hyperdense collection along the anterior margin of the C7 vertebral body.

Diagnostic evaluations included an EKG, a troponin assay, and a chest radiograph, all of which returned negative results for cardiac issues. Imaging revealed a wide mediastinum (Figure 1), prompting a CT with IV contrast. The CT scan demonstrated a large prevertebral soft tissue hematoma extending from C3 through the carina (Figure 2), accompanied by a rightward and anterior deviation of the airway. Additionally, a lobulated 9-mm hyperdense collection was identified along the anterior margin of the C7 vertebral body (Figure 3), suggesting active contrast extravasation within the prevertebral soft tissues immediately caudal to the C6−7 fusion construct in the surgical bed. A repeat chest radiograph confirmed the presence of a 10.3 cm mediastinum.

### 2.4. Differential Diagnosis

The alarming presentation of chest pain and diaphoresis led the medical team to consider cardiac ischemia as the primary diagnosis initially. However, subsequent evaluation, notably the chest radiograph, shifted the focus to the widening mediastinum, prompting consideration of various etiologies. Potential causes include thoracic aortic aneurysm or dissection, traumatic aortic rupture, cardiac tamponade, fractured ribs or thoracic vertebrae, mediastinitis, and pneumomediastinum. Though less likely due to the acute nature of this case, other differentials should include hilar lymphadenopathy (either infectious or malignant) and mediastinal masses such as lymphoma, seminoma, or thymoma. Each of these conditions necessitates careful consideration and diagnostic evaluation to identify the underlying pathology and guide appropriate management strategies accurately. However, it is crucial to prioritize the exploration of the most likely cause, such as in this case, where the patient had undergone neck surgery and likely had postoperative hemorrhage.

### 2.5. Management and Treatment

**Figure 4:**
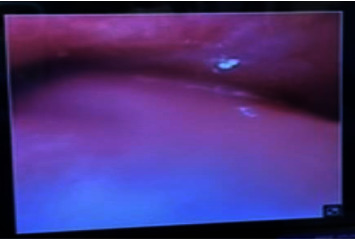
Bronchoscope image showing severe compression of the trachea before hematoma evacuation.

At the onset of symptoms 4 h postoperative, the patient was initially worked up for cardiac ischemia. Cardiothoracic surgery was consulted, and the patient was evaluated approximately 2 h after the onset of symptom. Given the emergent nature of their findings, the patient was transferred to the operating room for exploration and evacuation of the hematoma by neurosurgery almost 3 h after the patient complained of chest pain. Despite endotracheal intubation, ventilation was unsuccessful. Bronchoscopy revealed compression of the trachea, prompting immediate action (Figure 4). The neck was prepared with betadine, and an emergency neck exploration was performed through the incision made earlier by the neurosurgery team. Upon opening the platysma, a significant amount of fresh blood and hematoma was encountered, necessitating urgent evacuation. With successful hematoma evacuation, ventilation was restored.

On bronchoscopy, there was still posterior compression of the membranous distal airway and extensive mucosal edema. Utilizing the left cervical incision that was already made and the bronchoscope, neurosurgery, with the assistance of vascular surgery, bluntly dissected behind the airway and esophagus and directly onto the spine. The residual hematoma was bluntly loosened and evacuated. The remaining portion was explored, and some additional hematoma was carefully extracted. There was no vascular injury of a named vessel. Repeat bronchoscopy revealed some improvement in the distal airway patency, the remainder of which was likely related to the associated edema. Upper endoscopy was performed to ensure the esophageal mucosa was uninjured, and insufflated air under saline was used to confirm there was no extravasation of air. Once this was completed, hemostasis was confirmed, and the incision was closed with a drain.

### 2.6. Outcome and Follow-Up

On postoperative day (POD) 1, a chest radiograph revealed persistent widening of the upper mediastinum, and the patient remained intubated and sedated for airway protection. The next day, there was a significant improvement in hematoma size. A neck CT scan showed adequate drainage of hematoma and minimal soft tissue swelling without significant airway compression. Cardiac labs and exams were all normal. Extubation was planned for the next day, pending approval from the intensive care unit team, along with antibiotic therapy and prophylactic deep vein thrombosis management. The patient was successfully extubated on POD 5 with a stable neck wound and no bleeding. No other complaints were reported, and the patient was agreeable to physical and occupational therapy. Prophylactic anticoagulation was restarted. The patient was discharged from the hospital on POD 7, indicating successful recovery and resolution of the acute complication. At the 3-week follow-up appointment, the patient was noted to be doing well, with a chest radiograph showing no effusion or hematoma.

## 3. Discussion

Several risk factors contribute to the development of postoperative hematomas, including hypertension, smoking history, male sex, low BMI, long operative times, increased venous pressure, coagulopathy, multilevel surgery, and the invasiveness of the surgical procedure itself [6, 9]. The management of neck hematoma following cervical spine surgery necessitates proactive strategies for early recognition and intervention. Implementing protocols for rigorous postoperative monitoring is crucial, particularly for promptly identifying symptoms such as chest pain and respiratory distress indicative of hematoma formation. Equally vital is fostering a collaborative approach among surgical and critical care teams to ensure swift communication and coordinated responses to emergent situations. Continuous postoperative monitoring, including regular physical examinations and imaging studies, further enhances the ability to detect and manage complications effectively. These recommendations underscore the significance of timely intervention and multidisciplinary teamwork in optimizing patient outcomes.

Algorithms for the diagnosis and treatment of neck hematoma following cervical spine surgery should consider various factors, including clinical presentation, radiological findings, and patient comorbidities [5]. Signs such as worsening dyspnea, stridor, neck swelling, and neurological deficits typically occur within the first 6 h postoperative or up to 24 h and warrant urgent evaluation and intervention [10]. Diagnostic imaging modalities such as CT with IV contrast play a crucial role in confirming the diagnosis and assessing the extent of hematoma. Given the potential for disastrous airway compromise, protecting the airway becomes paramount, and alternative airway access with cricothyroidotomy and/or immediate evacuation of obstructive hematomas should be considered if intubation becomes difficult [11]. Therefore, having equipment such as a bronchoscope and a capable team available for immediate exploration is essential to facilitate prompt intervention and optimize patient outcomes. In a prior review of 785 anterior cervical discectomy and fusion procedures, nine cases (1.15%) of neck hematoma resulting in acute airway obstruction were observed. Notably, none of the nine patients had preoperative risk factors for hematoma; six of the nine patients developed a hematoma within 24 h of surgery [12]. These findings highlight the importance of maintaining a high level of clinical suspicion for hematoma and acute airway obstruction during the early postoperative period. Airway compromise can evolve from subtle changes in voice quality and complaints of difficulty talking and breathing during the early period to dyspnea, stridor, and cyanosis during later stages [13]. Critical airway compromise, consisting of the near or complete loss of airway patency, requires an emergent response progressing from bag-mask ventilation, to intubation, and surgical exploration and evacuation of the hematoma [13]. In cases where the airway cannot be re-established by these means, establishment of a surgical airway by cricothyroidotomy is required [13].

In this case, the decision to contact the primary neurosurgeon, along with cardiothoracic and vascular surgery consults, initiated a coordinated care approach aimed at addressing the emergent situation comprehensively. However, the initial focus on chest pain and cardiac ischemia led to a delay in accessing the operating room, potentially impacting the management of the hematoma. Had the neck examination been completed promptly by the primary surgeon, it is plausible that the hematoma might not have progressed to a stage necessitating the involvement of cardiothoracic and vascular surgery. This highlights the necessity of early physical examination for the evaluation of potential postoperative hematoma and for timely surgical intervention upon diagnosis. There are reported cases where hematomas mimic myocardial infarction, leading to the misdiagnosis and subsequent treatment of non-ST segment elevation myocardial infarctions, leading to detrimental consequences [14]. This diagnostic challenge highlights the importance of maintaining a broad differential diagnosis but, more importantly, prompt recognition of hematoma-related symptoms and examination. Fortunately, in the current case, the delayed diagnosis of neck hematoma did not result in an adverse outcome, as the patient ultimately recovered uneventfully and made a full return to normal activities.

Performing ACS surgeries in ambulatory surgical centers (ASCs) versus hospitals presents unique considerations regarding the management of complications, particularly hematoma formation. While ASCs offer advantages such as efficiency and cost-effectiveness [15, 16], they may lack the resources and infrastructure required to manage emergent situations such as hematoma-related airway compromise effectively [17]. Patients undergoing ACS procedures in ASCs may face delays in accessing specialized care and interventions, potentially exacerbating the risk of adverse outcomes associated with hematoma formation [18]. Therefore, careful patient selection, consideration of procedural complexity, and access to emergency care are essential when determining the appropriateness of ACS in ASCs, with a focus on ensuring timely access to comprehensive medical services and resources necessary for managing complications such as hematomas. Moreover, it is noteworthy that our case was managed in a hospital setting, which likely facilitated prompt access to specialized care and interventions, thus potentially avoiding additional delays compared to if the procedure had been performed at an ASC.

In conclusion, neck hematoma following cervical spine surgery is a rare but potentially life-threatening complication that requires prompt recognition and intervention. While cardiac issues are a valid concern, especially given the patient's symptoms, the markedly lower prevalence of cardiac complications underscores the need for early consultation, vigilant monitoring for signs of hematoma formation, and adherence to a structured algorithm for triage and management to ensure timely intervention and favorable outcomes in these challenging cases.

## Data Availability

Patient identifiers were removed, and thus, consent was not required. Due to patient confidentiality, medical records cannot be made public for review.

## References

[1] McCarthy M. H., Weiner J. A., Patel A. A. (2020). Strategies to Achieve Spinal Fusion in Multilevel Anterior Cervical Spine Surgery: An Overview. *HSS Journal*.

[2] Buttermann G. R. (2018). Anterior Cervical Discectomy and Fusion Outcomes Over 10 Years: A Prospective Study. *Spine*.

[3] Marquez-Lara A., Nandyala S. V., Fineberg S. J., Singh K. (2014). Incidence, Outcomes, and Mortality of Reintubation After Anterior Cervical Fusion. *Spine*.

[4] Miao W., Ma X., Liang D., Sun Y. (2018). Treatment of Hematomas After Anterior Cervical Spine Surgery: A Retrospective Study of 15 Cases. *Neurochirurgie*.

[5] Stirling D., Klar G., Franklin J., Mizubuti G. B. (2022). Complete Airway Obstruction and Massive Hemorrhage From Post-Thyroidectomy Neck Hematoma: A Case Report and Management Algorithm.

[6] Epstein N. (2020). Frequency, Recognition, and Management of Postoperative Hematomas Following Anterior Cervical Spine Surgery: A Review. *Surgical Neurology International*.

[7] Shah-Becker S., Greenleaf E. K., Boltz M. M., Hollenbeak C. S., Goyal N. (2018). Neck Hematoma After Major Head and Neck Surgery: Risk Factors, Costs, and Resource Utilization. *Head & Neck*.

[8] Fineberg S. J., Oglesby M., Patel A. A., Singh K. (2013). Incidence and Mortality of Perioperative Cardiac Events in Cervical Spine Surgery. *Spine*.

[9] Bovonratwet P., Fu M. C., Tyagi V. (2019). Incidence, Risk Factors, and Clinical Implications of Postoperative Hematoma Requiring Reoperation Following Anterior Cervical Discectomy and Fusion. *Spine*.

[10] Thakkar K., Nwangene N. L., Maharjan R. (2023). A Comprehensive Management of Neck Hematoma in Post-Thyroidectomy Patient for Papillary Thyroid Cancer: A Case Report. *Cureus*.

[11] O’Neill K. R., Neuman B., Peters C., Riew K. D. (2014). Risk Factors for Postoperative Retropharyngeal Hematoma After Anterior Cervical Spine Surgery. *Spine*.

[12] Song K.-J., Choi B.-W., Lee D.-H., Lim D.-J., Oh S.-Y., Kim S.-S. (2017). Acute Airway Obstruction Due to Postoperative Retropharyngeal Hematoma After Anterior Cervical Fusion: A Retrospective Analysis. *Journal of Orthopaedic Surgery and Research*.

[13] Palumbo M. A., Aidlen J. P., Daniels A. H., Thakur N. A., Caiati J. (2012). Airway Compromise Due to Wound Hematoma Following Anterior Cervical Spine Surgery. *The Open Orthopaedics Journal*.

[14] Ghanchi H., Siddiqi I., Takayanagi A., Patchana T., Fakhoury F. J. (2020). Spontaneous Spinal Subdural Hematoma Mimicking Myocardial Infarction. *Cureus*.

[15] Helseth Ø., Lied B., Halvorsen C., Ekseth K., Helseth E. (2015). Outpatient Cervical and Lumbar Spine Surgery Is Feasible and Safe: A Consecutive Single Center Series of 1449 Patients. *Neurosurgery*.

[16] McGirt M. J., Rossi V., Peters D. (2020). Anterior Cervical Discectomy and Fusion in the Outpatient Ambulatory Surgery Setting: Analysis of 2000 Consecutive Cases. *Neurosurgery*.

[17] Epstein N. E. (2016). Cervical Spine Surgery Performed in Ambulatory Surgical Centers: Are Patients Being Put at Increased Risk?. *Surgical Neurology International*.

[18] Sheha E. D., Derman P. B. (2019). Complication Avoidance and Management in Ambulatory Spine Surgery. *Journal of Spine Surgery*.

